# Quantitative Analysis of Temporal Bone Density and Thickness for Robotic Ear Surgery

**DOI:** 10.3389/fsurg.2021.740008

**Published:** 2021-09-30

**Authors:** Emile Talon, Miranda Visini, Franca Wagner, Marco Caversaccio, Wilhelm Wimmer

**Affiliations:** ^1^Hearing Research Laboratory, ARTORG Center for Biomedical Engineering Research, University of Bern, Bern, Switzerland; ^2^Department for Otolaryngology, Head and Neck Surgery, Inselspital University Hospital Bern, Bern, Switzerland; ^3^Department of Diagnostic and Interventional Neuroradiology, Inselspital, Bern University Hospital, Bern, Switzerland

**Keywords:** BAHA, bone conduction implants, screw safety, bone thickness, bone mineral density, calibrated Hounsfield units, quantitative computed-tomography

## Abstract

**Background and Objective:** Quantitative assessment of bone density and thickness in computed-tomography images offers great potential for preoperative planning procedures in robotic ear surgery.

**Methods:** We retrospectively analyzed computed-tomography scans of subjects undergoing cochlear implantation (*N* = 39). In addition, scans of Thiel-fixated *ex-vivo* specimens were analyzed (*N* = 15). To estimate bone mineral density, quantitative computed-tomography data were obtained using a calibration phantom. The temporal bone thickness and cortical bone density were systematically assessed at retroauricular positions using an automated algorithm referenced by an anatomy-based coordinate system. Two indices are proposed to include information of bone density and thickness for the preoperative assessment of safe screw positions (Screw Implantation Safety Index, SISI) and mass distribution (Column Density Index, CODI). Linear mixed-effects models were used to assess the effects of age, gender, ear side and position on bone thickness, cortical bone density and the distribution of the indices.

**Results:** Age, gender, and ear side only had negligible effects on temporal bone thickness and cortical bone density. The average radiodensity of cortical bone was 1,511 Hounsfield units, corresponding to a bone mineral density of 1,145 mg HA/cm^3^. Temporal bone thickness and cortical bone density depend on the distance from Henle's spine in posterior direction. Moreover, safe screw placement locations can be identified by computation of the SISI distribution. A local maximum in mass distribution was observed posteriorly to the supramastoid crest.

**Conclusions:** We provide quantitative information about temporal bone density and thickness for applications in robotic and computer-assisted ear surgery. The proposed preoperative indices (SISI and CODI) can be applied to patient-specific cases to identify optimal regions with respect to bone density and thickness for safe screw placement and effective implant positioning.

## 1. Introduction

In robotic ear surgery, high-resolution computed-tomography (CT) imaging has proven invaluable to evaluate the complex anatomy of the temporal bone and to ensure safe and effective surgical procedures. To avoid damage to at-risk anatomical structures, geometric information has been the focus of preoperative planning in computer-assisted otological microsurgery (1–6). Importantly, CT images can additionally provide information about bone density that could be utilized to infer on local bone strength for preoperative planning procedures related to robotic ear surgery. The temporal bone contains a variety of bone tissue ranging from pneumatized regions of low density (mastoid air cells) to regions with the highest density present in the human body (petrous bone). Uncalibrated CT radiodensity values expressed as Hounsfield units (HU) enable to study the maturation of temporal bone tissue (7). However, a verified correspondence between the indicated radiodensity and the actual bone mineral density requires the acquisition of calibrated CT images (8, 9). So-called quantitative CT imaging is commonly applied to diagnose and monitor osteoporosis (10), but so far only received limited attention in otology (11).

In robotic cochlear implantation, fiducial screws are implanted retroauricularly as artificial landmarks to achieve the required patient-to-image registration accuracy and to fix the dynamic reference base for tracking patient motion (12, 13). As a firm placement of the fiducial screws is crucial to guarantee safe procedures, the locations for screw insertion have to provide sufficient cortical layer thickness and surrounding bone density. To the best of our knowledge, the direct link between screw osseointegration and bone mineral density has not been specifically analyzed for the temporal bone. However, studies were performed for other regions: the direct relation between screw pullout strength and bone mineral density was verified in the lumbar spine (14) and orthopedic screw fixation was analyzed with respect to bone mineral density in a computational study (15). Fiducial screws placed inferiorly on the temporal bone often coincide with mastoid air cells causing reduced mechanical stability. Moreover, bone density is an important factor considered to minimize heat (16) and acoustic noise exposure during bone removal and drilling (17, 18). Firm screw placement is also desired for the immobilization of bone conduction, middle ear, or cochlear implant bodies, in particular in pediatric cases (19). For bone conduction implants, which exert vibrations to the bone to stimulate the inner ear, screws serve additionally as a means of sound transmission, making a firm placement particularly important, along with the implant location and coupling type (20–22). In the case of bone-anchored hearing aids long-term osseointegration is required for efficient sound transmission (23, 24). Furthermore, primary instability is one of the main causes for hearing implant failure, together with surgical errors (25). All these applications require finding optimal positions in terms of available bone thickness and density.

Therefore, the aim of this work was to quantitatively assess the temporal bone density and thickness in adult subjects for applications in robotic ear surgery. In addition, we propose radiograph-based indices for the preoperative assessment of implant body and screw locations for optimized screw stability and mass distribution in the temporal bone.

## 2. Materials and Methods

### 2.1. Study Design and Data Collection

We performed a retrospective analysis on clinical high-resolution CT scans (Somatom Definition Edge, Siemens, Germany; 94 mA, 120 kV, voxel size: 0.156 × 0.156 × 0.2 mm^3^) taken at the Department of Neuroradiology at the University Hospital in Bern between 2015 and 2017. In total, temporal bone scans of 39 subjects (17 female, 21 male; mean age 55 years, range 21 to 79 years) undergoing cochlear implantation were evaluated. No subjects with temporal bone malformations or osteoporosis were included in the analysis. In addition, to assess the influence of specimen preparation on bone densities, we included high-resolution temporal bone CT scans of Thiel-fixed whole head specimens (*N* = 19) (26) in the analysis.

### 2.2. Temporal Bone Segmentation and Surface Mesh

For each subject, the temporal bone was segmented using the open-source platform 3D Slicer (27). Bone structures were labeled for voxel intensities above a threshold of 620 HU [according to the compact bone threshold reported by (28)] in a region bounded anteriorly by the posterior wall of the external auditory canal, inferiorly by the tip of the mastoid process, posteriorly by the occipitomastoid suture, and superiorly by the temporal line. To obtain a uniform label structure and to account for the pneumatization of the temporal bone, the labels were post-processed by removing single islands containing less than 300 voxels. Using the labels, a three-dimensional surface mesh was generated using a marching cubes algorithm and smoothed with a kernel size of 4 mm. For the consecutive analysis, the DICOM data together with the surface meshes were imported into Matlab (The MathWorks Inc., Natick, MA, USA).

### 2.3. Retroauricular Coordinate System

**Figure 1:**
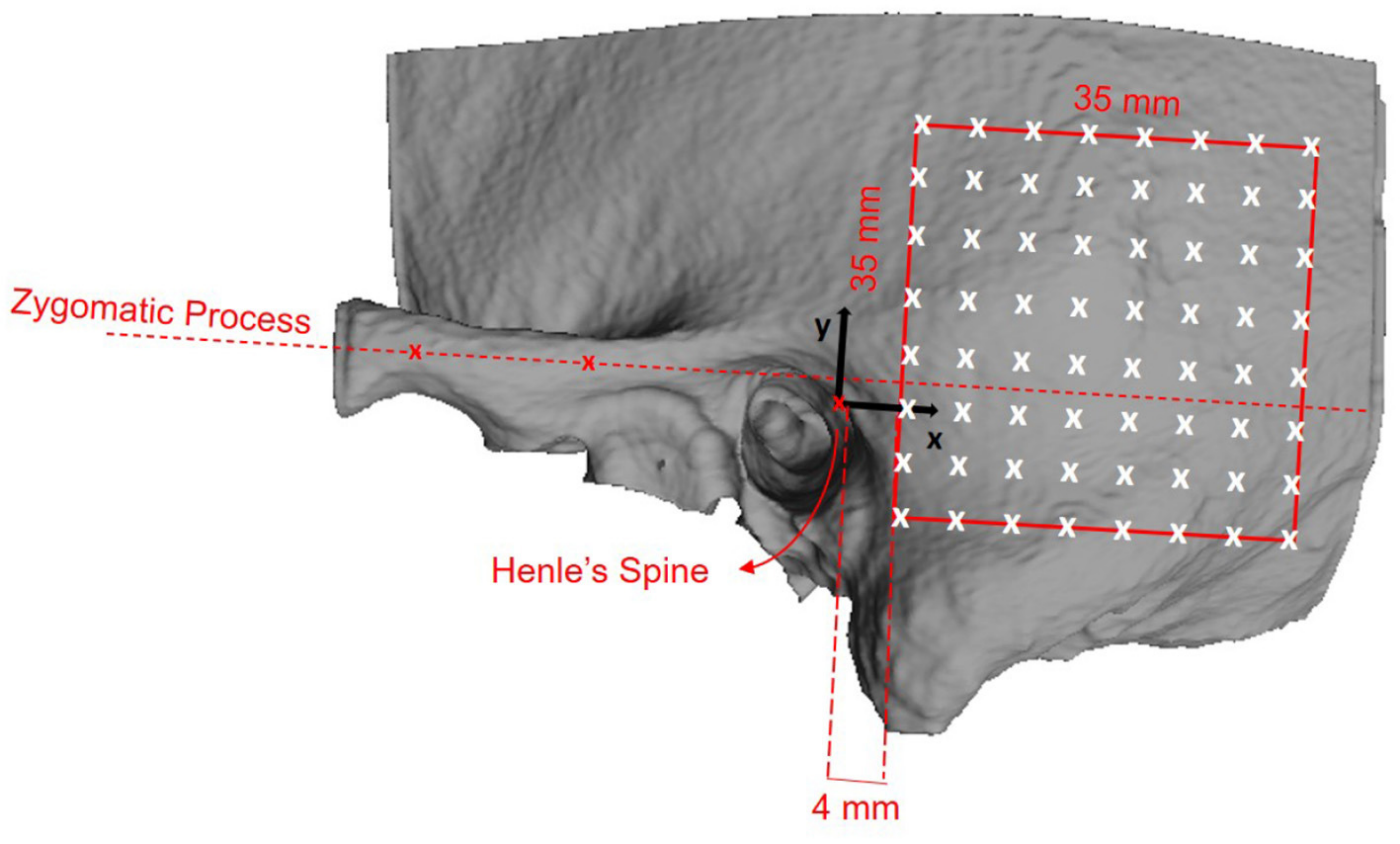
Definition of the retroauricular coordinate system and grid specification for the analyzed region of interest (ROI). The origin of the coordinate system lies at Henle's spine. The x-axis is oriented along the zygomatic process, as specified by two landmarks. The x/y-plane is perpendicular to the transversal image plane as defined by the clinical protocol. The red square indicates the ROI containing the 8 × 8 probe grid. The probe positions are equally spaced by a distance of 5 mm, resulting in a covered area of 35 × 35 mm^2^. The lower anterior corner of the ROI is positioned at x = 4 mm and y = −10 mm.

We defined a retroauricular coordinate system using anatomical landmarks that are easily and reliably identifiable during otological procedures (5, 29). With this approach, the surgeon can transfer preoperatively planned positions on the temporal bone using a ruler. The origin of the Cartesian coordinate system is defined by the most superior point on Henle's spine, while two manually selected points along the center of the zygomatic process specify the orientation of the x-axis. Using the coordinate system, a region of interest (ROI) with a grid of 64 probe positions was specified (Figure 1).

### 2.4. Bone Mineral Density Calibration

**Figure 2:**
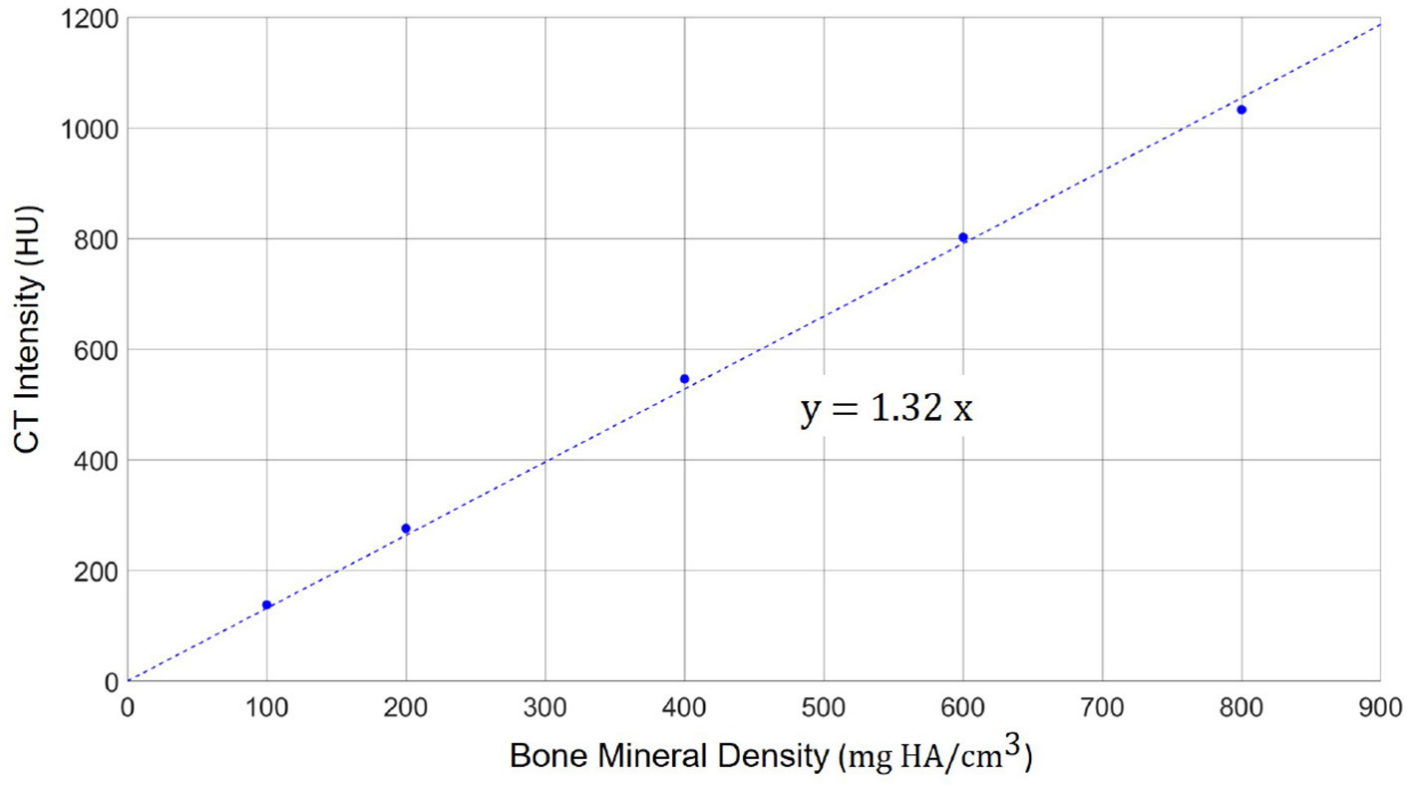
Calibration scale between the radiodensity in the applied high-resolution CT imaging protocol (in HU) and the actual bone mineral density. A linear relation can be observed with a scaling factor of 1.32 between HU and mg HA/cm^3^.

To enable a quantitative analysis of bone mineral density expressed as the concentration of hydroxyapatite (mg HA/cm^3^), we calibrated the radiodensity of the applied CT imaging protocol on the same scanner using a dedicated phantom (QRM-BDC-6, QRM GmbH, Moehrendorf, Germany). The phantom contains 6 cylindrical inserts providing references for 0 HU (water), as well as 100, 200, 400, 600, and 800 mg HA/cm^3^. The obtained calibration graph shows a linear relation between radiodensity (HU) and bone mineral density (9) (Figure 2). Negative values of HU were set to 0 in order to obtain only positive values of the calibrated bone mineral density.

### 2.5. Evaluation of Bone Thickness and Cortical Density

**Figure 3:**
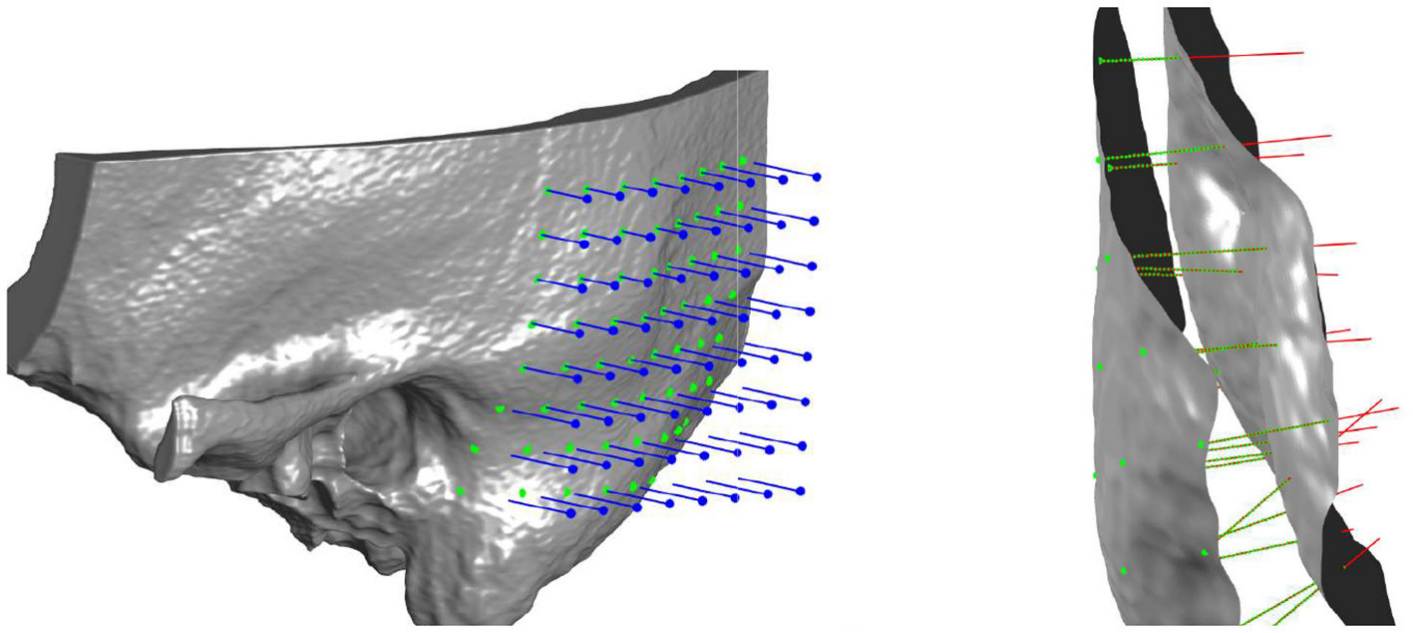
**Left**: Three-dimensional visualization of the projection of grid points onto the outer surface of the temporal bone mesh. **Right**: Probe evaluation in a section of the temporal bone. Blue lines represent the probes that from the mask intercept the outer bone surface. Red lines represent the normal direction to the external surface, along which thickness and densities are computed. In green are highlighted the points where density is measured for every probe, spaced between each other by 0.15 mm.

**Figure 4:**
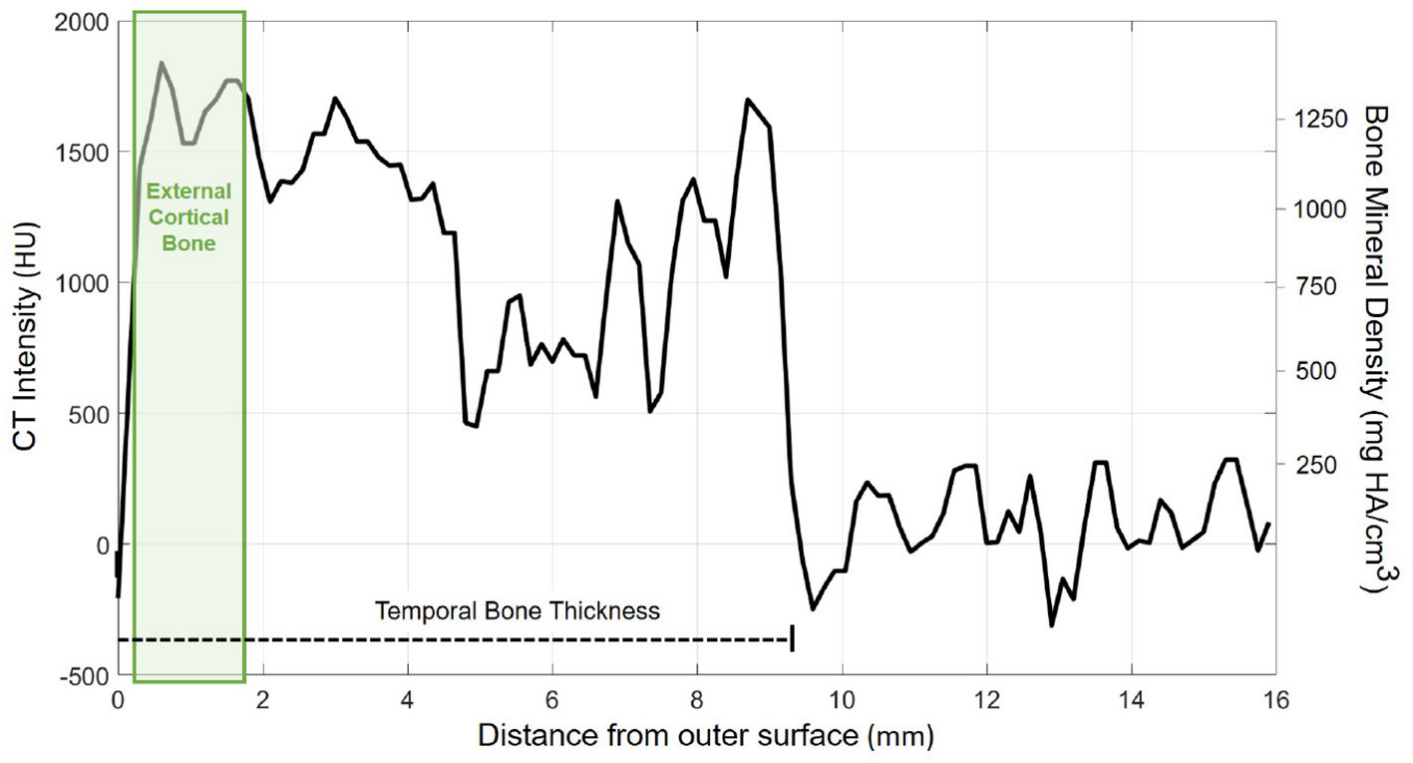
Exemplary course of radiodensity (in HU) and bone mineral density (in mg HA/cm^3^) along a probe trajectory. The temporal bone thickness (d_TB_) along the trajectory is indicated by a dashed line. The green shaded area indicates the region considered for external cortical bone density computation.

The probe positions of the grid were projected onto the surface mesh along the surface normal of the x/y-plane (Figure 3, left). Every probe (blue line in Figure 3, right) intersects the temporal bone mesh in a specific point (highlighted in green) that lays inside one of the triangles of the mesh. This point is then the origin of a trajectory normal to the triangle surface. Consecutively, the intensity values (in HU) of voxels intersecting each trajectory were extracted (Figure 3, right). An example of the extracted intensity profile along a trajectory is shown in Figure 4. Using the surface mesh, the temporal bone thickness (d_TB_) was defined as the distance from the start position to the last intersected triangle on the opposite surface. The maximum bone thickness was limited to 18 mm. For the computation of the external cortical bone density, the intensity values were averaged over a thickness of 1.5 mm, starting from the first point along the trajectory with a radiodensity of at least 1,000 HU (see Figure 4), as suggested by (7).

### 2.6. Preoperative Planning Indices

#### 2.6.1. Screw Implantation Safety Index (SISI)

To assess the level of safety for the implantation of screws in the temporal bone, e.g., surgical fiducial screws in robotic ear surgery or for implant fixation, we propose the Screw Implantation Safety Index (SISI). The SISI considers both, the available bone thickness and the bone density along the probe trajectory. First, to avoid interference of the screw with soft tissue, a bone thickness threshold (d_min_) is specified. In our analysis, d_min_ was chosen with 4 mm and 5 mm according to screw lengths commonly used in ear surgery. Probe locations that have a smaller bone thickness than the required threshold have a SISI of 0. Locations with sufficient bone thickness (at least d_min_ mm) are considered for the next computation step. To compute the SISI (in %), the number of sampled voxels with a radiodensity of at least 1,000 HU (30) are counted (N_S_) and divided by the total number of sampled voxels (N) present within the thickness threshold (d_min_) along the probing trajectory:


$$\text{SISI}=\{\begin{array}{ll} \frac{{\text{N}}_{\text{S}}}{\text{N}}\;\cdot 100 & \;{\text{d}}_{\text{TB}}>{\text{d}}_{\text{min}}\\ 0 & \;{\text{d}}_{\text{TB}}\leq {\text{d}}_{\text{min}}. \end{array}$$

#### 2.6.2. Column Density Index (CODI)

To provide quantitative information about bone mass distribution in the temporal bone, we propose a second index, the Column Density Index (CODI). It is defined as the sum of the bone mineral density values measured along the probing trajectory for the full temporal bone thickness (d_TB_). The CODI represents a mass per unit surface area, also called column density (expressed in mg HA/mm^2^):


$$\begin{array}{l} \text{CODI}=\overset{\text{N}}{\underset{i=0}{\sum }}\rho_{\text{TB}}\left(i\right)\cdot \Delta \text{d}, \end{array}$$

where N denotes the total number of sampled voxels along the probing trajectory (and within d_min_), ρ_TB_(*i*) is the bone mineral density for each sampled voxel (in mg HA/mm^3^), and Δd is the sampling interval along the trajectory (in our case 0.15 mm).

### 2.7. Statistical Analysis

Differences in bone thickness, cortical bone density, as well as the SISI and CODI indices were estimated using separate linear mixed-effects models, with fixed effects for the retroauricular coordinates in the x and y directions (in mm), age (in years) gender (female vs. male), and ear side (left vs. right). A subject-level random effect was included to account for paired measurements. A significance level of 0.05 was used for all comparisons. The statistical analysis was performed using R Studio and the “lme4” package (31).

## 3. Results

### 3.1. Temporal Bone Thickness

**Figure 5:**
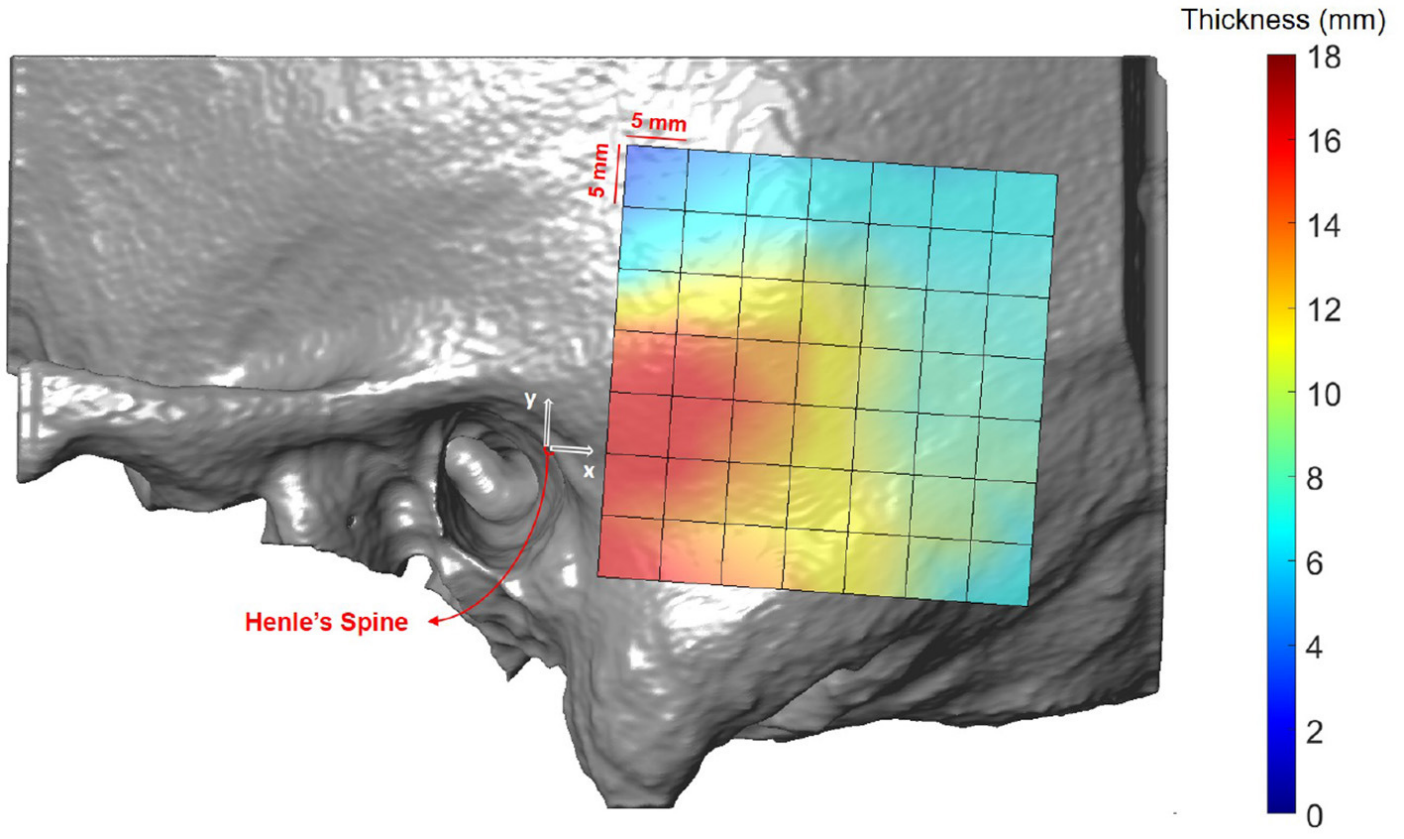
Heat map visualization of temporal bone thickness in the retroauricular region of interest averaged across all subjects.

Figure 5 illustrates the temporal bone thickness averaged across all subjects, excluding *ex-vivo* samples. The corresponding numerical values for the retroauricular grid can be found in Supplementary Table 1. The temporal bone is known to be thicker in the sinodural angle, becoming thinner superior to the lateral skull base and posterior to the occipitomastoid suture (5, 29). On average, the bone thickness decreases by 0.16 mm (*p* < 0.001) and 0.19 mm (*p* < 0.001) per millimeter distance from the origin (Henle's spine) in the x and y directions, respectively. Neither age (*p* = 0.25), gender (*p* = 0.54), nor ear side (*p* = 0.46) had a statistical significant effect on the bone thickness in our data (see Supplementary Table 2).

### 3.2. Cortical Bone Density

**Figure 6:**
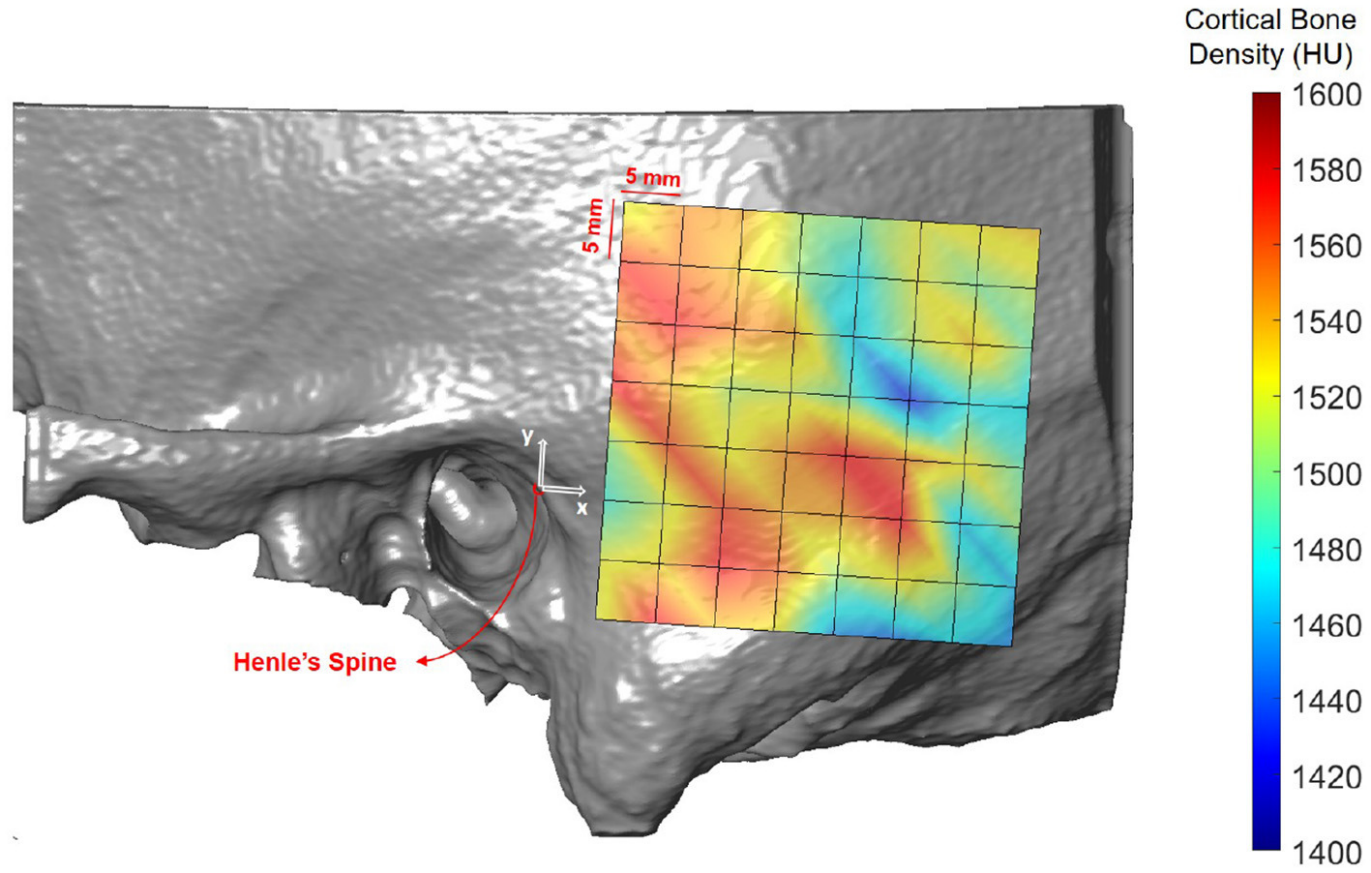
Heat map visualization of cortical bone density (in HU) in the retroauricular region of interest averaged across all subjects.

**Figure 7:**
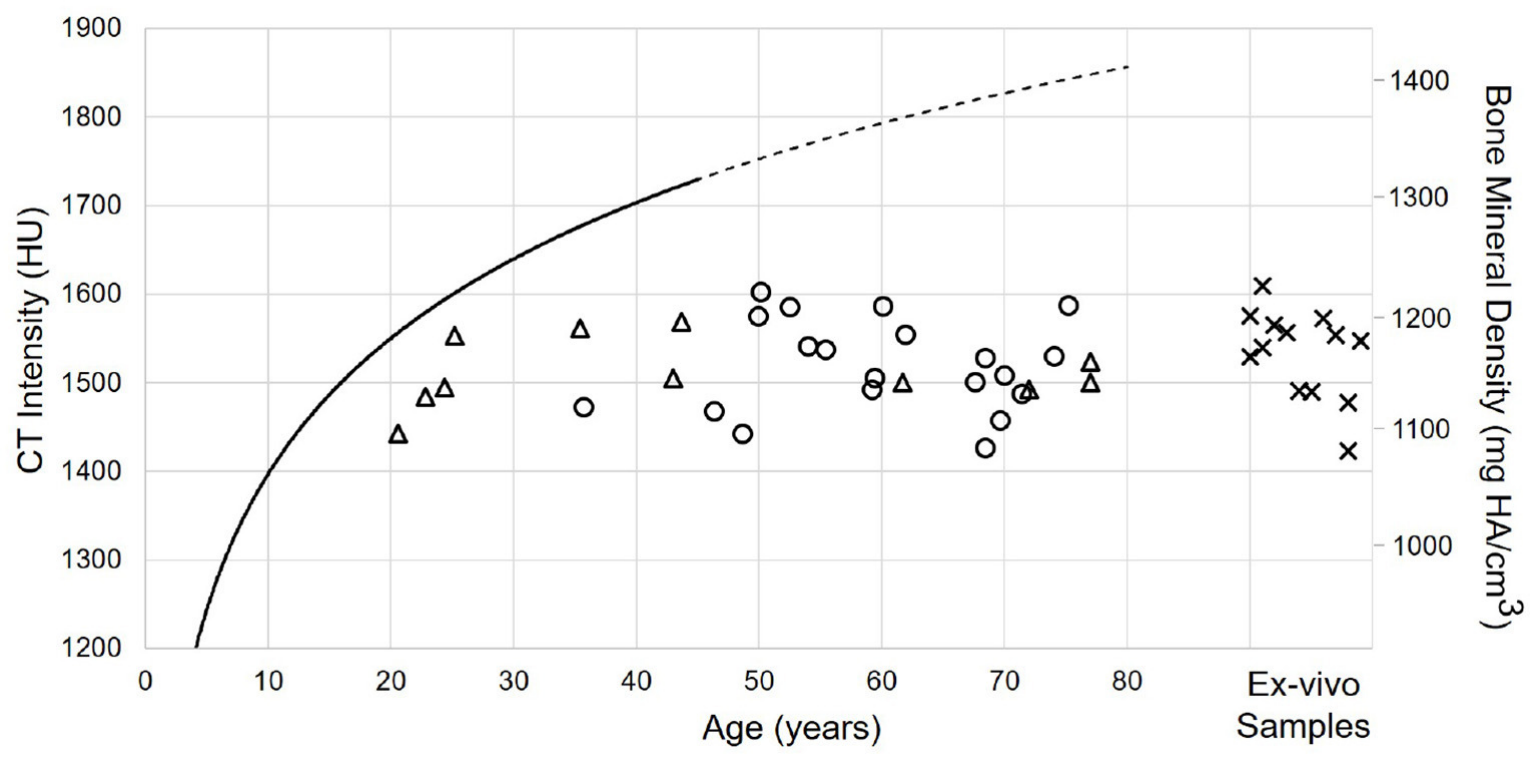
Relation between age and cortical bone density for male (circles) and female subjects (triangles), as well as Thiel fixed *ex-vivo* specimens (crosses). The solid black line indicates the model described by (7). The line is dashed for the prediction of the model.

Figure 6 shows the spatial distribution of cortical bone density across the temporal bone. The average radiodensity was 1511 HU (standard deviation: 241 HU), corresponding to a bone mineral density of 1145 mg HA/cm^3^. Age (*p* = 0.52) and gender (*p* = 0.72) did not have an effect on bone density (see Supplementary Table 4), while right ear sides tended to have slightly smaller densities (difference 47 HU; *p* = 0.03) The cortical bone density did not change significantly along the y-axis (*p* = 0.30), however, it reduced by 1.8 HU (*p* < 0.001) per millimeter distance along the x-axis. The relation between the average cortical bone density of individual subjects and age is provided in Figure 7. For comparison, the bone density development curve of (7) is also plotted.

### 3.3. Screw Implantation Safety Index (SISI)

**Figure 8:**
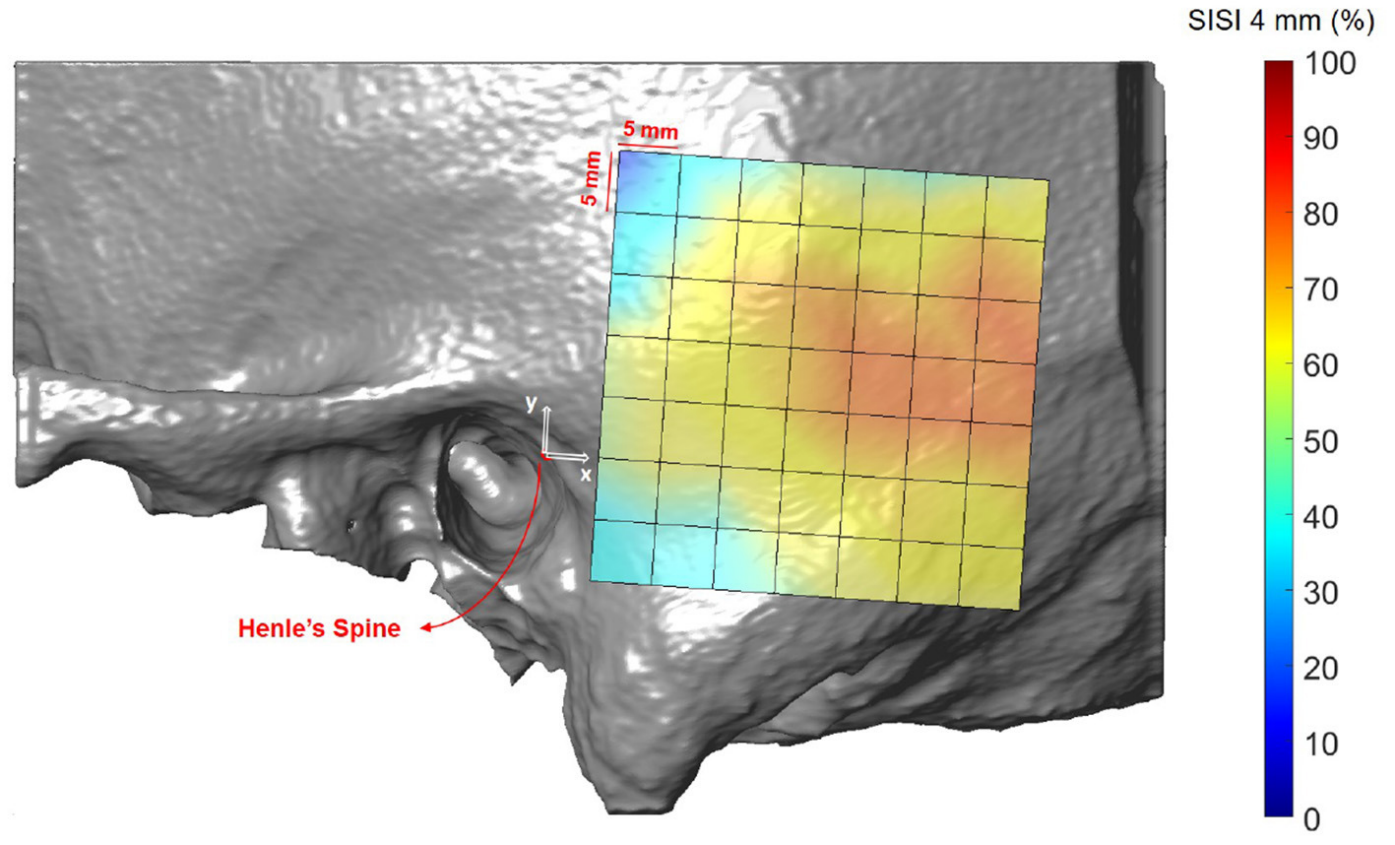
Visualization of the screw implantation safety index (SISI) for a screw length of 4 mm in the region of interest, averaged across all subjects.

**Figure 9:**
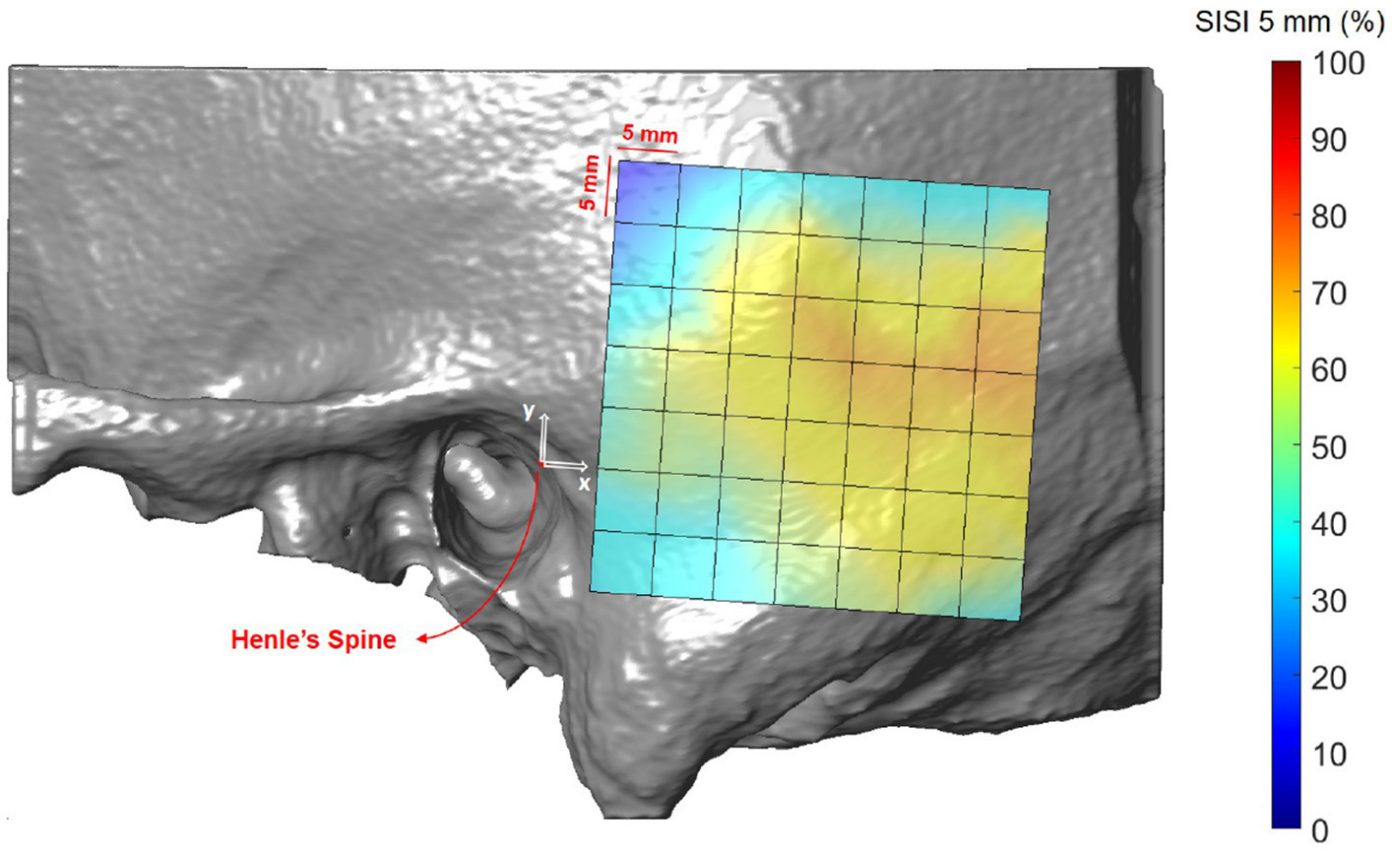
Visualization of the screw implantation safety index (SISI) for a screw length of 5 mm in the region of interest, averaged across all subjects.

Figures 8, 9 illustrate the spatial distribution of the SISI calculated for 4 and 5 mm screw lengths, respectively. The distributions are similar, with generally higher values for the SISI for the 4 mm screw lengths, as these require less bone thickness. Neither ear side, age nor gender had an effect on SISI 4 and 5 values. For both indices, higher values were observed on average for increasing distances along the x direction, where variations along the y direction had less influence on the SISI (see Supplementary Tables 6, 8). In regions closer to Henle's spine, the higher occurrence of mastoid air cells is reflected in generally lower SISI values, although the temporal bone has a greater thickness (see Figure 5).

### 3.4. Column Density Index (CODI)

**Figure 10:**
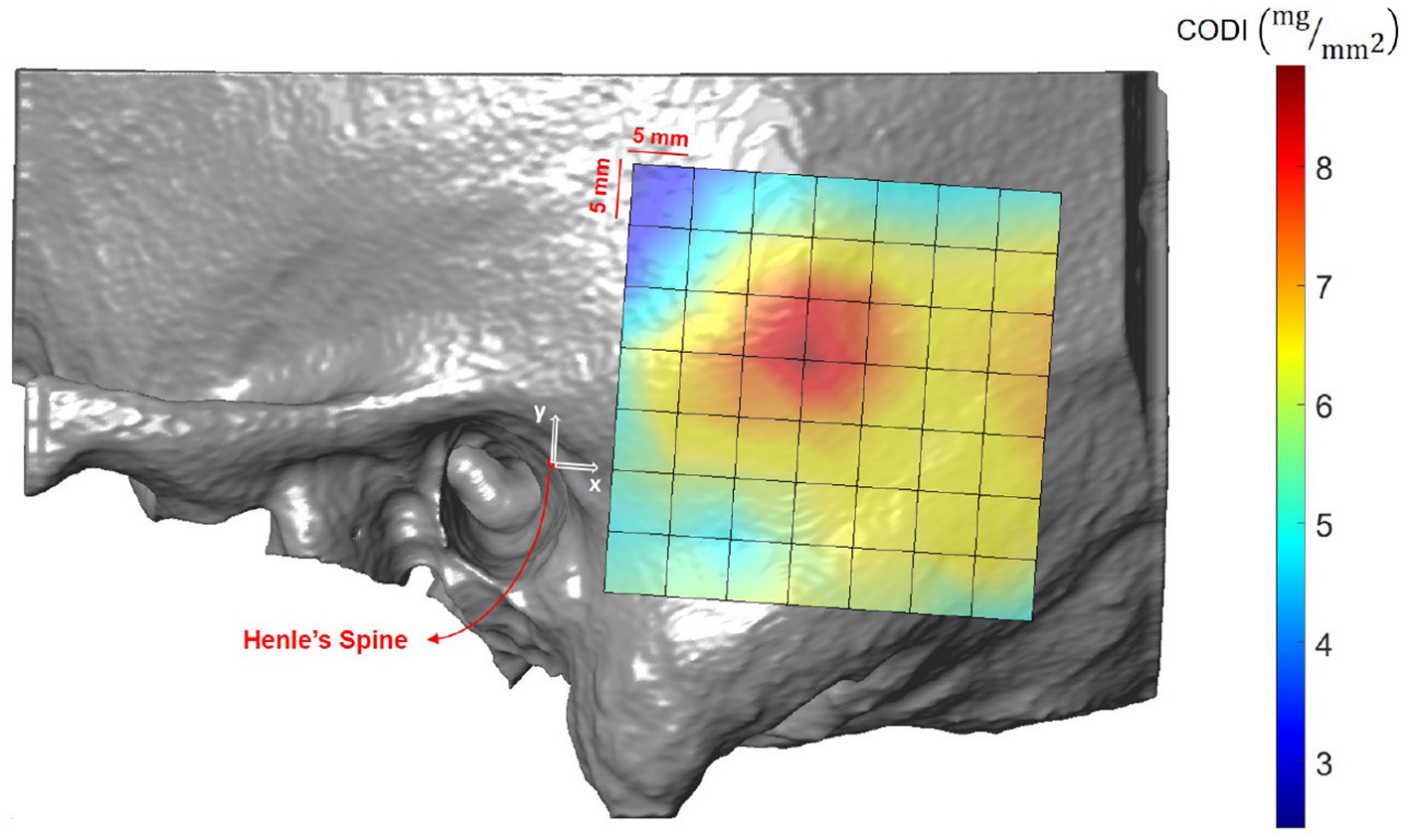
Visualization of the column density index (CODI) in the region of interest, averaged across all subjects.

Results averaged across all the subjects (excluding *ex-vivo* samples) are shown in Figure 10 and summarized in Supplementary Table 9. In the region posterior to the supramastoid crest (i.e., at positions 19 mm along the x-axis and 10 mm along the y-axis) the highest column densities were observed, indicating a local concentration of bone mass.

## 4. Discussion

Minimally invasive robot-assisted ear surgery relies on preoperative planning procedures to identify landmarks for patient image registration and to plan access routes at safe distances from structures at risk. Obviously, assessment of geometric properties, particularly available bone thickness, is central to screw and implant placement. The presented study highlights novel aspects that include bone density in the preoperative planning phase. We show how information about radiodensity (or calibrated bone mineral density) can be used to provide a refined assessment of the local bone situation and associated mechanical strength properties. We introduced quantitative CT imaging, i.e., the assessment of calibrated bone mineral density, to the domain of computer-assisted otological planning procedures. Quantitative CT imaging offers several interesting applications for preoperative assessment, e.g., for the classification of otosclerotic cases (32). We applied a clinically motivated reference frame in the retroauricular region to allow coordinate transfer by identifying anatomic landmarks *in situ* and using rulers, in case of preparatory steps are required (e.g., fiducial screw placement) or no navigation system is available. Other transfer methods, such as template-guided approaches (33), could also be used. For patient-specific planning, the proposed methods and indices could be computed using automated segmentation tools (34, 35).

### 4.1. Temporal Bone Thickness

Temporal bone thickness has been extensively studied in the context of otological surgery (5, 34, 36–38). Our study reproduces the known variability, showing the largest available bone thickness within a radius of 19 mm from *Henle's* Spine. As expected, temporal bone thickness is not age dependent in adult subjects. For bone-anchored hearing aids, the suggested screw implantation position is limited in proximity to the auditory ear canal to avoid contact with the pinna. The implantation site commonly used in bone-anchored hearing aids is located at a distance of 45–50 mm to Henle's spine and 30° inclination with respect to the zygomatic process (39). In our reference frame, this corresponds to positions at x = 39 mm and y = 20–25 mm. In these locations, the observed thickness varied from 5.9 to 7.0 mm, which is sufficient to host a 4 mm implant without damaging underlying soft tissue (39). One limitation of our study is the limited sample size. Studies including additional data need to be performed to test if our findings can also be reproduced in larger cohorts. As our data set does not include pediatric cases, no conclusions about children (exhibiting significantly lower bone thickness) can be drawn (40). Children exhibit smaller temporal bones (41), and screw lengths of 4 mm are usually only applicable at age 6 or older, while screw lengths of 3 mm often are only possible at age 2 or older. This highlights also the limitation of application of fiducial screws for robotic ear surgery in very young subjects or subjects with temporal bone malformations.

### 4.2. Cortical Bone Density

The distribution of cortical bone density can be considered rather uniform within and in-between subjects, regardless of age, gender, ear side or preservation of specimens. This is in accordance to (7), who analyzed the maturation of bone density in different regions in the temporal bone, such as the lateral surface or the otic capsule. The overall higher densities in the model of (7) can be explained by the differences in the applied assessment methods. (7) used a two-dimensional approach in single CT slices with small probing areas (0.3 mm^2^) to avoid partial volume effects. In addition, their study included subjects aged 3 months to 42 years. In contrast, in this study, the cortical bone density was computed as the average along a 1.8 mm thick probing trajectory, including also less dense regions. Future studies could include data from subjects younger than 20 years to provide a comparable measure in the maturation of the cortical bone density. Moreover, larger, age-matched data sets are required to validate our findings.

### 4.3. Screw Implantation Safety Index

The first preoperative planning index that we propose is the Screw Implantation Safety Index (SISI). It could provide guidelines to surgeons for patient-specific screw placement in otological surgery. Herein, we analyzed the SISI for 4 and 5 mm screw lengths, which are dimensions typically used for implants (e.g., bone-anchored hearing aids) or fiducial screws used in robotic ear surgery. However, the index can be adapted to other screw dimensions. As shown in Figures 8, 9, the visualization of the SISI using a heat map provides an intuitive representation of bone density and thickness to identify optimal regions for screw placement. Our results indicate that ideal locations for screw placement on the temporal lie within 24–39 mm posteriorly and 5–15 mm superiorly to Henle's spine. Optimal implantation locations for 5 mm screws are located approximately 25 mm posterior to Henle's spine.

### 4.4. Column Density Index (CODI)

As a second preoperative indicator, we propose the column density index (CODI) quantifying the amount of bone mass in the temporal bone. The main motivation behind the CODI is to identify suitable regions for efficient coupling of bone conduction implants (20). For example, the local concentration of mass posterior to the supramastoid crest could be preferred for fixation, as more mass should result in more efficient coupling and sound transmission. The local maximum results from the contribution of two parameters: temporal bone thickness and the presence of mastoid air cells.

## 5. Conclusions

This study applied a combined assessment of temporal bone density and thickness to provide novel perspectives for the preoperative planning in robotic ear surgery. Quantitative verification of the proposed indices related to mechanical properties requires further evaluation with larger sample size, including biomechanical testing.

## Data Availability Statement

The original contributions presented in the study are included in the article/Supplementary Material, further inquiries can be directed to the corresponding author/s.

## Ethics Statement

Data collection and analysis for this study was approved by the local institutional review board (reference numbers 2017-01462 and 2016-00887).

## Author Contributions

ET and WW: conceptualization. ET, FW, and WW: methodology. ET, MV, and WW: formal analysis. FW and MC: resources. ET: writing/original draft preparation. MV, FW, MC, and WW: writing/review and editing. MC and WW: funding acquisition. All authors contributed to the article and approved the submitted version.

## Funding

This work was funded by the Bern University Hospital (Inselspital).

## Conflict of Interest

The authors declare that the research was conducted in the absence of any commercial or financial relationships that could be construed as a potential conflict of interest. The handling editor PH declared a past co-authorship with the authors MC and WW.

## Publisher's Note

All claims expressed in this article are solely those of the authors and do not necessarily represent those of their affiliated organizations, or those of the publisher, the editors and the reviewers. Any product that may be evaluated in this article, or claim that may be made by its manufacturer, is not guaranteed or endorsed by the publisher.
