# Supplementary material for: Quantitative Analysis of Temporal Bone Density and Thickness for Robotic Ear Surgery
Source: Front Surg. 2021 Sep 30;8:740008. doi: 10.3389/fsurg.2021.740008 (PMC8514837; doi:10.3389/fsurg.2021.740008)
Supplement: Supplementary file 1 [file Data_Sheet_1.PDF]

## SUPPLEMENTAL DATA

**Table 1.** Temporal bone thickness (in mm) averaged across all subjects (excluding ex-vivo specimens) for the retroauricular grid as defined in Figure 1.

| | ref + 4mm | ref + 9mm | ref + 14mm | ref + 19mm | ref + 24mm | ref + 29mm | ref + 34mm | ref +39mm |
|---|---|---|---|---|---|---|---|---|
| **ref + 25mm** | 2.71 | 3.76 | 4.73 | 5.39 | 5.45 | 5.20 | 5.63 | 5.87 |
| **ref + 20mm** | 3.72 | 5.35 | 6.67 | 7.11 | 7.37 | 6.88 | 6.78 | 7.04 |
| **ref + 15mm** | 6.38 | 9.28 | 10.41 | 10.65 | 9.48 | 7.84 | 7.67 | 7.89 |
| **ref + 10mm** | 14.34 | 15.06 | 13.78 | 13.42 | 10.48 | 8.33 | 8.04 | 7.79 |
| **ref + 5mm** | 15.97 | 16.64 | 15.37 | 13.45 | 10.01 | 8.25 | 8.37 | 8.05 |
| **ref** | 16.77 | 16.45 | 14.18 | 12.51 | 10.51 | 9.04 | 8.73 | 8.64 |
| **ref - 5mm** | 15.30 | 14.24 | 12.94 | 12.17 | 11.76 | 9.76 | 9.21 | 7.27 |
| **ref - 10mm** | 16.47 | 16.06 | 14.56 | 13.64 | 11.56 | 9.48 | 7.10 | 5.87 |

**Table 2.** Linear mixed-effects model results for temporal bone thickness.

| | Estimate | Std. Error | $p$ value |
|---|---|---|---|
| **(Intercept)** | 13.23 | 1.76 | $< .001^{***}$ |
| **Gender** | 0.57 | 0.92 | .54 |
| **Ear Side** | -0.61 | 0.83 | .46 |
| **Age** | -0.03 | 0.03 | .25 |
| **Direction $x$** | -0.17 | 0.007 | $< .001^{***}$ |
| **Direction $y$** | -0.19 | 0.007 | $< .001^{***}$ |

**Table 3.** Cortical density (in HU) averaged across all subjects (excluding ex-vivo specimens) for the retroauricular grid as defined in Figure 1.

| | ref + 4mm | ref + 9mm | ref + 14mm | ref + 19mm | ref + 24mm | ref + 29mm | ref + 34mm | ref +39mm |
|---|---|---|---|---|---|---|---|---|
| **ref + 25mm** | 1518.13 | 1551.53 | 1545.54 | 1508.47 | 1496.26 | 1520.80 | 1532.63 | 1540.48 |
| **ref + 20mm** | 1571.53 | 1555.95 | 1538.30 | 1503.87 | 1474.92 | 1520.50 | 1498.06 | 1512.48 |
| **ref + 15mm** | 1549.23 | 1576.42 | 1554.69 | 1546.49 | 1463.43 | 1509.62 | 1539.70 | 1493.55 |
| **ref + 10mm** | 1577.48 | 1541.27 | 1507.78 | 1513.59 | 1492.33 | 1428.42 | 1476.09 | 1467.92 |
| **ref + 5mm** | 1501.76 | 1566.14 | 1524.26 | 1550.57 | 1577.40 | 1554.14 | 1528.32 | 1490.81 |
| **ref** | 1482.40 | 1514.24 | 1567.66 | 1545.17 | 1546.57 | 1580.75 | 1462.73 | 1502.29 |
| **ref - 5mm** | 1517.93 | 1533.92 | 1576.76 | 1560.50 | 1505.51 | 1527.73 | 1502.46 | 1458.84 |
| **ref - 10mm** | 1516.19 | 1579.21 | 1539.04 | 1529.23 | 1451.59 | 1443.58 | 1466.11 | 1447.37 |

**Table 4.** Linear mixed-effects model results for the cortical bone density.

| | Estimate | Std. Error | $p$ value |
|---|---|---|---|
| **Intercept** | 1510.5 | 46.0 | $< .001^{***}$ |
| **Gender** | 8.5 | 23.7 | 0.72 |
| **Ear Side** | 47.0 | 21.0 | $0.031^{*}$ |
| **Age** | 0.5 | 0.7 | 0.52 |
| **Direction $x$** | -1.8 | 0.3 | $< .001^{***}$ |
| **Direction $y$** | 0.4 | 0.4 | 0.3 |

**Table 5.** Screw Implantation Safety Index (SISI) for 4 mm screws, averaged across all subjects (excluding ex-vivo specimens) for the retroauricolar grid as defined in Figure 1. Results are shown in percentage.

| SISI 4 mm | ref + 4mm | ref + 9mm | ref + 14mm | ref + 19mm | ref + 24mm | ref + 29mm | ref + 34mm | ref +39mm |
|---|---|---|---|---|---|---|---|---|
| **ref + 25mm** | 12.77 | 32.66 | 40.91 | 51.30 | 49.03 | 45.24 | 51.39 | 57.97 |
| **ref + 20mm** | 24.98 | 40.32 | 62.31 | 66.22 | 62.31 | 65.09 | 73.77 | 67.18 |
| **ref + 15mm** | 32.24 | 54.99 | 73.12 | 74.70 | 74.70 | 72.04 | 80.30 | 77.17 |
| **ref + 10mm** | 53.67 | 60.71 | 67.63 | 71.99 | 80.08 | 79.34 | 77.64 | 81.29 |
| **ref + 5mm** | 52.41 | 58.74 | 64.38 | 71.71 | 78.38 | 80.02 | 80.41 | 79.16 |
| **ref** | 50.97 | 57.89 | 57.33 | 61.37 | 69.45 | 70.85 | 68.62 | 71.96 |
| **ref - 5mm** | 37.74 | 44.45 | 43.52 | 57.24 | 57.61 | 69.59 | 67.86 | 66.78 |
| **ref - 10mm** | 33.59 | 39.85 | 41.64 | 47.84 | 56.20 | 57.44 | 56.74 | 56.51 |

**Table 6.** Linear mixed-effects model for the screw implantation safety index for 4 mm screws. Results are expressed in percentage.

| | Estimate | Std. Error | *p* value |
|---|---|---|---|
| **Intercept** | 37.92 | 7.86 | $< .001^{***}$ |
| **Gender** | -3.49 | 4.06 | 0.40 |
| **Ear Side** | 2.30 | 3.65 | 0.533 |
| **Age** | 0.07 | 0.12 | 0.573 |
| **Direction $x$** | 0.89 | 0.053 | $< .001^{***}$ |
| **Direction $y$** | 0.11 | 0.053 | $0.034^{*}$ |

**Table 7.** Screw Implantation Safety Index (SISI) for 5 mm screws, averaged across all subjects (excluding ex-vivo specimens) for the retroauricolar grid as defined in Figure 1. Results are shown in percentage.

| SISI 5 mm | ref + 4mm | ref + 9mm | ref + 14mm | ref + 19mm | ref + 24mm | ref + 29mm | ref + 34mm | ref +39mm |
|---|---|---|---|---|---|---|---|---|
| **ref + 25mm** | 7.49 | 16.32 | 27.86 | 37.74 | 38.87 | 33.13 | 33.96 | 33.09 |
| **ref + 20mm** | 16.92 | 29.71 | 50.27 | 61.62 | 50.71 | 51.85 | 52.23 | 65.49 |
| **ref + 15mm** | 25.82 | 37.53 | 64.71 | 71.24 | 66.67 | 61.76 | 72.99 | 72.61 |
| **ref + 10mm** | 43.24 | 56.80 | 61.88 | 68.73 | 74.31 | 72.38 | 75.98 | 74.05 |
| **ref + 5mm** | 44.04 | 53.41 | 57.28 | 65.65 | 68.37 | 70.67 | 68.72 | 70.85 |
| **ref** | 46.18 | 51.70 | 49.46 | 54.72 | 66.33 | 68.11 | 62.59 | 68.82 |
| **ref - 5mm** | 33.15 | 37.15 | 39.63 | 52.55 | 55.19 | 63.91 | 60.88 | 53.44 |
| **ref - 10mm** | 29.81 | 35.68 | 39.71 | 45.36 | 51.63 | 54.42 | 44.07 | 40.24 |

**Table 8.** Linear mixed-effects model for the screw implantation safety index for 5 mm screws. Results are expressed in percentage.

| | Estimate | Std. Error | *p* value |
|---|---|---|---|
| **Intercept** | 29.02 | 9.53 | $.004^{**}$ |
| **Gender** | -2.43 | 4.93 | .63 |
| **Ear Side** | 1.13 | 4.43 | .80 |
| **Age** | 0.15 | 0.15 | .32 |
| **Direction $x$** | 0.81 | .058 | $< .001^{***}$ |
| **Direction $y$** | -0.08 | 0.058 | .18 |

**Table 9.** Column Density Index (CODI, in mg HA/mm$^2$) averaged across all subjects (excluding ex-vivo specimens) for the retroauricular grid as defined in Figure 1.

| | ref + 4mm | ref + 9mm | ref + 14mm | ref + 19mm | ref + 24mm | ref + 29mm | ref + 34mm | ref +39mm |
|---|---|---|---|---|---|---|---|---|
| **ref + 25mm** | 2.44 | 3.30 | 4.16 | 4.99 | 4.70 | 4.72 | 4.99 | 5.14 |
| **ref + 20mm** | 2.78 | 4.04 | 5.72 | 6.15 | 6.07 | 5.93 | 6.08 | 6.37 |
| **ref + 15mm** | 3.49 | 5.29 | 7.20 | 8.08 | 7.59 | 6.61 | 6.75 | 7.14 |
| **ref + 10mm** | 5.42 | 6.86 | 7.67 | 8.85 | 7.69 | 6.70 | 6.91 | 7.19 |
| **ref + 5mm** | 5.16 | 6.82 | 7.20 | 7.13 | 6.88 | 6.20 | 6.54 | 7.11 |
| **ref** | 5.38 | 5.95 | 5.94 | 6.07 | 6.52 | 6.38 | 6.58 | 6.78 |
| **ref - 5mm** | 4.10 | 5.23 | 4.85 | 5.72 | 6.33 | 6.22 | 6.63 | 6.04 |
| **ref - 10mm** | 5.44 | 5.61 | 5.74 | 6.17 | 5.91 | 5.82 | 5.29 | 5.10 |

**Table 10.** Linear mixed-effects model for the Column Density Index (CODI).

| | Estimate | Std. Error | *p* value |
|---|---|---|---|
| **Intercept** | 4.73 | 1.19 | $< .001^{***}$ |
| **Gender** | -0.78 | 0.62 | 0.22 |
| **Ear Side** | 0.1 | 0.56 | 0.86 |
| **Age** | 0.016 | 0.018 | 0.38 |
| **Direction *x*** | 0.039 | 0.005 | $< .001^{***}$ |
| **Direction *y*** | -0.011 | 0.005 | .02* |
